# Mechanisms of High Concentration Valine-Mediated Inhibition of Peach Tree Shoot Growth

**DOI:** 10.3389/fpls.2020.603067

**Published:** 2020-10-29

**Authors:** Suhong Li, Futian Peng, Yuansong Xiao, Qingtao Gong, Ziyi Bao, Yanyan Li, Xuelian Wu

**Affiliations:** ^1^State Key Laboratory of Crop Biology, College of Horticulture Science and Engineering, Shandong Agricultural University, Tai’an, China; ^2^Shandong Institute of Pomology, Tai’an, China

**Keywords:** peach trees, valine, *PpSnRK1*, *PpTOR*, branched chain amino acids

## Abstract

The vigorous growth of the new shoots of the peach tree was not beneficial to high quality and efficient cultivation. High concentration of amino acids can inhibit plant growth, but the mechanism is not clear. In this study, we explored the regulatory effects of seven amino acids (phenylalanine, valine, leucine, isoleucine, serine, D-alanine, and proline) (10 g⋅L^–1^) on the growth of peach trees. The results showed that phenylalanine, valine, and proline inhibited peach seedling growth and valine has the most significant effect and it can promote the root growth of peach seedlings. Compared with paclobutrazol, valine treatment improves net photosynthetic rate and fruit quality without reducing shoot diameter or puncture strength, and it does not affect leaf morphology. Valine enhanced the expression of *PpSnRK1* (sucrose non-fermenting-1-related protein kinase) and inhibited the expression of *PpTOR* (Target of Rapamycin) and *PpS6K* (Ribosomal S6 kinase). The gibberellin content was significantly reduced in the valine treatment group. The endogenous valine content of peach seedlings was increased, acetohydroxyacid synthase (AHAS, E.C. 2.2.1.6) activity was inhibited by feedback, isoleucine synthesis was decreased, the relative amounts of branched chain amino acids were unbalanced, and growth was inhibited. However, isoleucine spraying after valine treatment could increase the content of isoleucine and alleviate the inhibition of valine on the shoot growth. In conclusion, valine is environmentally friendly to inhibit the growth of new shoots of peach trees by regulating the balance of *PpSnRK1* and *PpTOR* and the synthesis of isoleucine.

## Introduction

The vigorous growth of the new shoots of the peach tree leads to labor-consuming and time-consuming for plastic trimming, and is not conducive to the improvement of the ventilation and light transmission conditions of the tree (Hao, 2017). How to control the growth of new shoots effectively is very important to regulate the relationship between vegetative growth and reproductive growth of peach. Currently, paclobutrazol is the most widely used treatment for inhibiting new shoot growth. However, paclobutrazol treatment concentration is difficult to control, the levels of soil and fruit residues are high, and leaf shape, fruit shape index, and fruit tree disease resistance are affected, causing the banning of its application on fruit trees in some countries and regions (Huang et al., 1991; Xu, 1991; Yu and Song, 1997; Shalini and Sharma, 2006; Song et al., 2011; Li, 2013; Rong, 2017).

By contrast, amino acids are mainly obtained from plants, animal hair and industrial residues. The secondary utilization can not only be used as fertilizer to regulate plant growth, but also relieve environmental pressure. Moreover, previous studies in our laboratory showed that too high or too low concentration of amino acids (1–50 g⋅L^–1^) would not produce obvious stress effect on plant growth. Amino acids are promoters and catalysts for the synthesis of various enzymes in plants playing an important role in plant metabolism and having the advantages of being safe and environmentally friendly. At present, past studies of the effects of amino acids have mainly focused on plant growth promotion (He et al., 2019; Kitir et al., 2019; Wang et al., 2019), whereas less is known regarding their roles in plant growth inhibition (Forsum et al., 2008; Näsholm et al., 2009; Xu et al., 2018).

Valine, leucine, and isoleucine all contain special branched structures and are collectively called branched amino acids (BCAAs). Acetohydroxyacid synthase (AHAS, E.C. 2.2.1.6) is a key BCAAs enzyme that catalyzes the first biosynthetic step (Singh and Shaner, 1995; Duggleby et al., 2008). Acetohydroxyacid synthase is controlled by substrate specificity and subject to BCAAs-mediated feedback inhibition (Chipman et al., 2005; Lee et al., 2011). The response of AHAS to individual BCAAs is different in different organisms. In bacteria and yeast, AHAS is primarily inhibited by valine (Holmberg and Petersen, 1988). In barley, AHAS is inhibited by both leucine and valine, whereas valine can alleviate leucine-mediated AHAS inhibition in oat (Harris, 1956). By contrast, AHAS is not subject to any BCAAs feedback inhibition in mung bean (Satyanarayana and Radhakrishnan, 1964). The growth of *Arabidopsis thaliana* plants was shown to be significantly inhibited when supplied with valine as a nitrogen source (Forsum et al., 2008). Primary root growth in wild-type and AHAS-mutant *Arabidopsis* is inhibited under valine treatment, with increasing valine treatment concentration accompanied by an increasing degree of growth inhibition (Zhao et al., 2015).

*SnRK1* (sucrose non-fermenting-1-related protein kinase) and *TOR* (Target of Rapamycin) are two important “Yin-Yang” regulatory hubs in plant growth (Margalha et al., 2019). Appropriate concentrations of amino acids can activate *TOR* and its downstream transcription and translation links (Kimball and Jefferson, 2006). However, the effect of high concentration of valine on *SnRK1*, *TOR* and *S6K* (Ribosomal S6 kinase) has not been reported.

Previous studies showed that high concentration amino acid could significantly inhibit the growth of plant. However, the potential inhibitory effect of amino acid on growth in peach trees, and its molecular mechanism, have not yet been reported. Following a study of the inhibitory effects of various amino acids on peach seedling growth, valine was selected for further analysis in this study. We found that 10g⋅L^–1^ valine inhibited the activity of AHAS, decreased isoleucine synthesis, and unbalanced the relative levels of branched chain amino acids. It also increased the expression of *PpSnRK1*, reduced the expression of *PpTOR* and *PpS6K*, reduced GA_3_ (gibberellin) content, inhibited peach tree new shoot growth, shortened internode length, and had no adverse effects on stem diameter or leaf and fruit quality. The results of this study provide a reference for the management of closely planted dwarf peach trees, ensuring their high yield and fruit quality. Moreover, these data provide new insights and methods for plant growth regulation through amino acid treatments.

## Materials and Methods

### Test Design

This experiment was carried out at the experimental base of the South University of Shandong Agricultural University in 2018–2019. It located in Tai’an City, Shandong Province, China. The annual average temperature is 13°C, the highest temperature in July is 26.4°C, and the lowest in January, with an average of -2.6°C. The average annual precipitation is 697 mm. Plump and uniform seeds of *Prunus persica* (L.) Batsch were soaked in gibberellin and then seeded in a seedling tray. When peach seedlings had grown to about 5 cm in height, seedlings of similar size with no diseases or pests were selected and planted in basins. Each basin was cylindrical, with an inner diameter of 20.8 cm and a height of 22 cm. The culture medium in the basin was a mix of garden soil, and vermiculite in a 2:1 ratio by volume. The weight of the medium in each basin was 1.5 kg. The seedlings received conventional management. The following nine treatments were imposed: phenylalanine (Phe), valine (Val), leucine (Leu), isoleucine (Ile), serine (Ser), D-alanine (D-Ala), proline (Pro), paclobutrazol (Pcba), and clear tap water (Control). The concentration of each amino acid (Shanghai Yuanye Biotechnology Co., Ltd.) was 10 g⋅L^–1^, and that of paclobutrazol (15% wettable powder, Sichuan Guoguang Agrochemical Co., Ltd.) was 5 g⋅L^–1^. Each treatment was replicated three times. Seedlings were sprayed once every three days, two times in total. The amino acids that were most effective for the growth inhibition of peach seedlings were identified and further to explore the mechanism of valine-mediated inhibition of peach seedlings.

The second experimentation was performed at the Hong Miao experimental base of Shandong Agricultural University from January to September 2019. Three-year-old Ruiguang-39 peach trees, which had a row spacing of 2 m × 5 m and displayed similar growth and plant vigor, were used as the experimental materials. During the study, routine management was carried out. Experimental treatments were performed in the morning or evening in clear and calm weather. Treatments were sprayed until both sides of the leaf were covered with beads of water but not dripping. Tree crowns were treated with 10 g⋅L^–1^ valine (Val) (Shanghai Yuan ye Biotechnology Co., Ltd.) and compared with those treated with 5 g⋅L^–1^ 15% paclobutrazol wettable powder (Pcba) (Sichuan Guoguang agrochemical Co., Ltd.) or clear water (Control). Three biological repetitions were included per treatment, and duplicate spray treatments were performed 3 days apart.

### Test Items and Methods

#### Height of Peach Seedlings (New Shoots) and Related Shoot Indices

The height of each peach seedling was measured from its base to its growing point using a rice ruler. Ten peripheral shoots at a tree height of 1–1.5 m and with comparable growth (about 5 cm) were randomly selected around the tree for marking. The length from the base of new shoots to the growing point was measured every 10 days. When the growth of new shoots in each treatment was measured twice to be less than or equal to 0.2 cm, the experiment was stopped.

The puncture strength was measured by a stem strength meter (DDY-1, Beijing Shun Ke Da science and Technology Co., Ltd.) at a distance of 10 cm from the base of new shoots. The diameter was measured with vernier caliper at a distance of 1 cm from the base of new shoots. The length of two internodes in the middle of new shoots was measured with a meter scale, and their average value was calculated to determine the internode length.

#### Leaf-Related Physical Parameters

The fifth and sixth uppermost leaf from the top of the plant was selected and fully unfolded. The photosynthetic rate was measured using a a ciras-3 portable photosynthetic instrument (PPSystems, United Kingdom).

A portable leaf area-measuring instrument (YMJ-A, Zhejiang Tuo Pu Yun Nong Technology Co., Ltd.) was used to measure leaf vertical diameter, transverse diameter, and area. Leaf dry weight was determined by drying leaves in an oven at 105°C for 30 min followed by incubation at 60°C until a static dry weight was attained. Leaf water content was determined by calculating the difference between leaf fresh and dry weights. Photos were taken to observe leaf shape.

The total N content was determined by Kjeldahl. The total P content was determined by vanadium molybdate yellow colorimetric. The total K content was determined with MODEL410 blaze photometer (Sherwood scientific, Britain). The total K content was determined by high-performance liquid chromatography. The soluble sugar was determined by the anthrone reagent (Zhao and Cang, 2015). The free amino acids were extracted and determined following the manufacturer’s instructions of an automatic amino acid analyzer (Biochrom, United Kingdom) (Lu and Lu, 2018).

#### Fruit-Related Parameters

Ten mature fruits that developed normally and were free of disease and insect pests were randomly selected from the middle of each tree crown to analyze fruit quality. The single fruit weight was measured using a balance. The vertical and transverse diameters were measured using a vernier caliper and used to calculate fruit shape index. The titratable acid content was determined by acid-base titration. The Vitamin C (Vc) content was determined by iodine titration (Zhao and Cang, 2015). The soluble solid content was determined with a TD-45 sugar analyzer. The ratio of sugar to acid was calculated. The sugar and organic acids components were extracted with ultrapure water and determined with capillary electrophoresis (Zhang et al., 2016).

#### Acetylhydroxylate Synthetase Activity

Activity of acetylhydroxylate synthetase (AHAS) was determined using an AHAS enzyme activity test kit (Shanghai Qi Yi Biotechnology Co., Ltd.) as follows: 0.2 g of fresh sample was weighed and frozen with liquid nitrogen, then ground and allowed to thaw at room temperature. Sample was added to extraction solution, mixed well, extracted at room temperature for 2 h, and then centrifuged. The supernatant (10 μL) was added to the reagents of the AHAS enzyme activity test kit and the OD value at 450 nm was measured.

#### RNA Extraction and qPCR Analysis

The sample RNA was extracted using an RNA Extraction Kit (Kang Wei Century Technology Co., Ltd.), and the cDNA for real-time fluorescent quantitative PCR was obtained using a Reverse Transcription Kit (Perfect Real Time, TaKaRa). The primers (Table 1) were designed using Primer 5.0 software. The Cfx96 Touch Real-Time PCR Detection System (Bio-Rad, United States) was used for real-time fluorescence quantitative PCR. Three biological and three technical replicates were performed for all qPCR reactions, and relative gene expression was calculated by the 2^–Δ^ ^Δ^ ^*CT*^ method with *PpActin* as the reference gene (Wang, 2014).

**TABLE 1:** Effects of valine and paclobutrazol treatments on exterior quality of peach fruit.

Treatment	Single fruit weight (g)	Vertical diameter (mm)	Transverse diameter (mm)	Fruit shape index
Val	290.06 ± 2.85c	80.23 ± 1.28b	83.51 ± 0.39b	1.02 ± 0.02a
Pcba	245.93 ± 2.75a	78.67 ± 1.28ab	78.60 ± 0.84a	1.04 ± 0.01a
Control	263.41 ± 2.63b	76.10 ± 0.91a	82.00 ± 2.20b	1.02 ± 0.01a

*Data are presented as means ± SD (*n* = 3). Different lowercase letters indicate statistically significant difference between values in the same column at the 0.05 level.*

### Data Processing and Analysis

SPSS 19.0 software was used to perform one-way analysis of variance, and differences among individual means were assessed using Duncan’s multiple range test (*P* < 0.05).

## Results and Analysis

### Effects of Different Amino Acids on the Growth of Peach Seedlings

**FIGURE 1:**
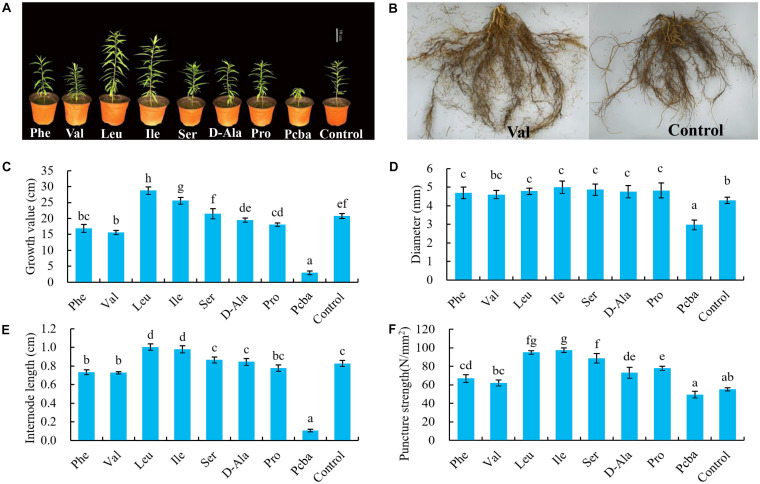
Effects of amino acid treatments on the **(A)** growth, **(B)** root system, **(C)** growth value, **(D)** diameter, **(E)** internode length, and **(F)** puncture strength of peach seedling. Bars indicate means ± SE (*n* = 3). Different letters on the bars indicate significant difference between the treatments.

Eighty days after the treatment, the height growth of peach seedlings was the greatest in leucine and the smallest in paclobutrazol (Figures 1A,C). Treatments phenylalanine, valine, proline, and paclobutrazol all had significant inhibitory effects on the growth of peach seedlings. With the exception of paclobutrazol, valine had the greatest inhibitory effect. Leucine and isoleucine had significant growth promoting effects. Although serine and D-alanine had inhibiting or promoting effects, they did not differ significantly from control. In conclusion, valine was the most effective amino acid for growth inhibition. Moreover, valine significantly shortened internode length without reducing stem diameter and puncture strength (Figures 1D–F). Also, spraying with valine promoted the root growth of peach seedlings, whereas paclobutrazol inhibited root growth (Figure 1B).

### Effects of Valine on Peach Tree New Shoot Growth and Leaf Morphology in Peach

**FIGURE 2:**
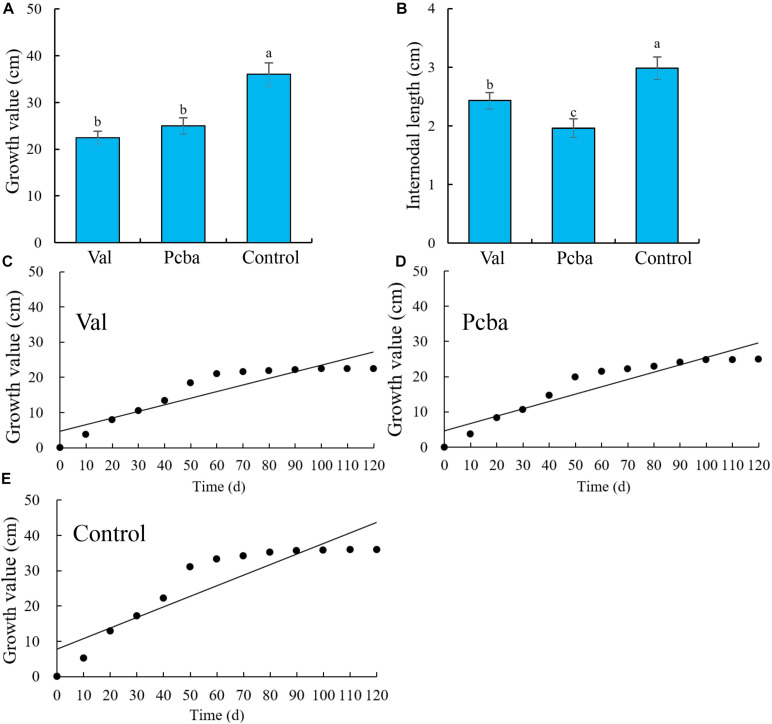
Effects of valine and paclobutrazol treatments on the new shoot **(A)** growth value, **(B)** internode length, and **(C–E)** growth dynamic in the treatment of valine, paclobutrazol, and water spraying of mature peach trees “Ruiguang-39.” Bars indicate means ± SE (*n* = 3). Different letters on the bars indicate significant difference between the treatments.

Following 120 days post-treatment growth, new shoot growth inhibition was observed in the order of valine < paclobutrazol < control. Valine and paclobutrazol significantly inhibited the growth of new shoots and resulted in significantly reduced internode lengths (Figure 2).

**FIGURE 3:**
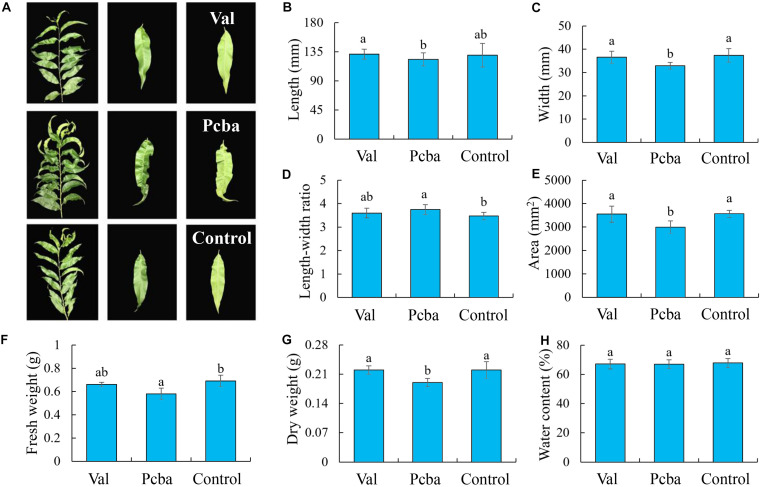
Effects of valine and paclobutrazol treatments on leaf **(A)** parameters, **(B)** length, **(C)** width, **(D)** length–width ratio, **(E)** area, **(F)** fresh weight, **(G)** dry weight, and **(H)** water content of mature peach trees “Ruiguang-39.” Bars indicate means ± SE (*n* = 3). Different letters on the bars indicate significant difference between the treatments.

Valine treatment had no significant effect on the analyzed leaf parameters, whereas paclobutrazol treatment resulted in significantly reduced leaf width, area, dry weight and fresh weight, and significantly increased aspect ratio (Figures 3B–G). Leaf water content remained unchanged by the different treatments (Figure 3H). Through observing leaf blade shape (Figure 3A), it was found that paclobutrazol treatment caused the leaf blade to shrink. These data indicate that valine treatment has no significant effect on peach tree leaf morphology.

### Effects of Valine on Peach Tree Net Photosynthetic and the Soluble Sugar and Mineral Element Accumulation

**FIGURE 4:**
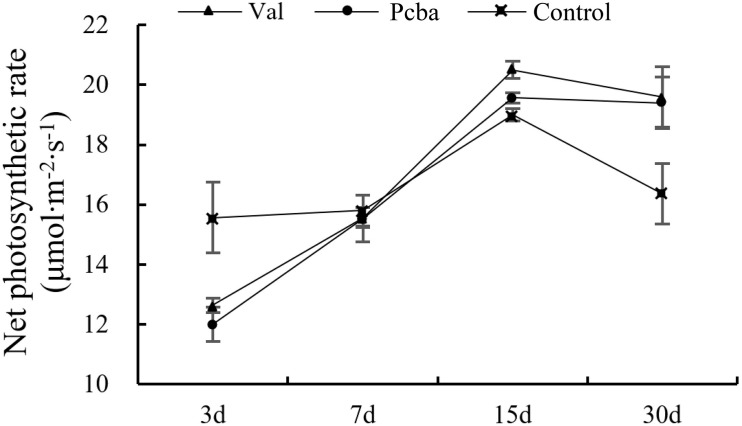
Effects of valine and paclobutrazol treatments on new net photosynthetic of mature peach trees “Ruiguang-39.” Each data point represents the mean (with SD bar) of three replicates.

At 3 days after valine treatment and paclobutrazol treatment, the net photosynthetic of treated plants were all significantly lower than that of the control plants. After that, the net photosynthetic increased and returned to the control level at 7th day and were significantly higher in valine-treated and paclobutrazol-treated plants at 15th and 30th days (Figure 4).

**FIGURE 5:**
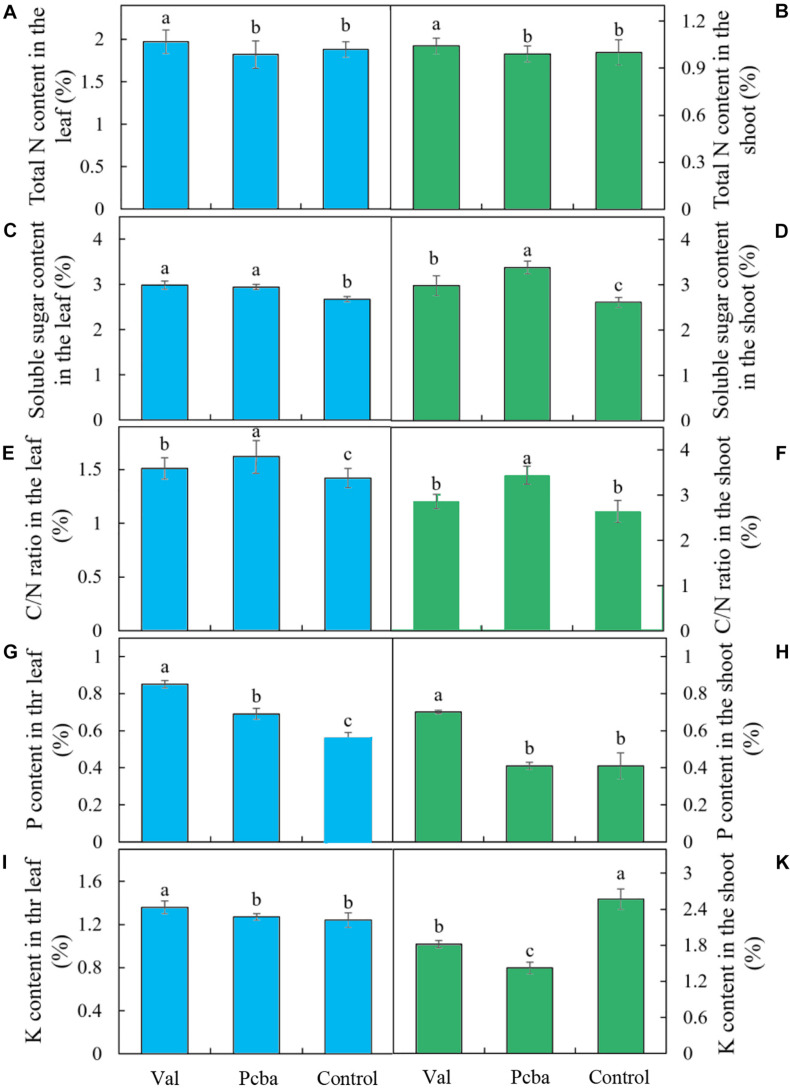
Contents of N, soluble sugar, P, K, and C/N ratio in the leaf **(A,C,E,G,I)** and shoot **(B,D,F,H,K)** of mature peach trees “Ruiguang-39.” Bars indicate means ± SE (*n* = 3). Different letters on the bars indicate significant difference between the treatments.

Compared with control, the contents of N, P, K, soluble sugar in valine-treated leaves and N, P, soluble sugar in valine-treated shoots increased significantly, whereas paclobutrazol treatment had no significant effect on the contents of N, K in leaves and N, P in new shoots. Moreover, K content in paclobutrazol-treatment shoots significantly lower than that of the control plants and valine-treated plants (Figure 5).

### Effects of Valine on Fruit Quality of Peach Trees

Compared with control, valine treatment significantly increased fruit weight and vertical diameter, whereas paclobutrazol treatment significantly reduced fruit weight and transverse diameter. Neither valine nor paclobutrazol treatment had a significant effect on the fruit shape index. These data indicate that valine treatment causes significantly increased single fruit weight and improved fruit appearance (Table 1).

Compared with control, valine significantly increased peach fruit soluble sugar contents, titratable acid, and Vc, which specifically were 14.9, 5.7, and 10.8% higher, respectively. Moreover, the two experimental treatments both resulted in reduced fruit hardness. These data indicate that valine treatment improves the internal quality of peach fruit by increasing soluble sugar, titratable acid, and Vc contents (Table 2).

**TABLE 2:** Effects of valine and paclobutrazol treatments on interior quality of peach fruit.

Treatment	Soluble Solid Content (%)	Soluble Sugar Content (mg/g)	Titratable Acid Content (mg/g)	Sugar/Acid	Vc Content (mg/g)	Hardness (kg/cm^2^)
Val	12.10 ± 0.29a	75.06 ± 1.86b	1.12 ± 0.05c	66.97 ± 2.60a	0.61 ± 0.05bc	13.55 ± 1.04a
Pcba	11.75 ± 0.20a	66.79 ± 0.84a	0.96 ± 0.05a	69.66 ± 3.32a	0.58 ± 0.04ab	14.23 ± 1.89a
Control	11.62 ± 0.18a	65.34 ± 2.54a	1.06 ± 0.04b	62.06 ± 4.95a	0.55 ± 0.01a	20.18 ± 1.60b

*Data are presented as means ± SD (*n* = 3). Different lowercase letters indicate statistically significant difference between values in the same column at the 0.05 level.*

**FIGURE 6:**
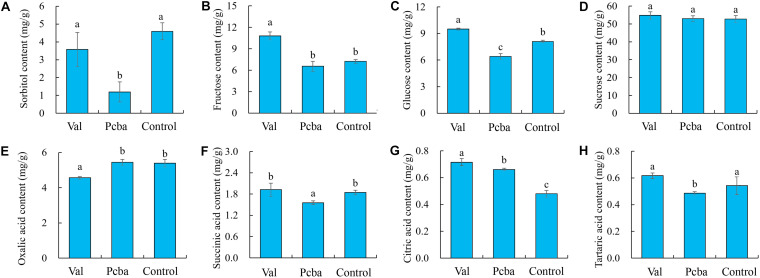
Effects of valine and paclobutrazol treatments on peach fruit sugar and acid components. **(A)** sorbitol, **(B)** fructose, **(C)** glucose, **(D)** sucrose, **(E)** oxalic acid, **(F)** succinic acid, **(G)** citric acid, **(H)** tartaric acid. Bars indicate means ± SE (*n* = 3). Different letters on the bars indicate significant difference between the treatments.

Compared with control, valine treatment significantly increased fructose, glucose and citric content, whereas paclobutrazol treatment significantly reduced sorbitol, glucose, succinic and tartaric content (Figure 6).

### Effect of Valine Treatment on the Expression of *PpSnRK1*, *PpTOR* and *PpS6K* Genes and GA_3_ Content

**FIGURE 7:**
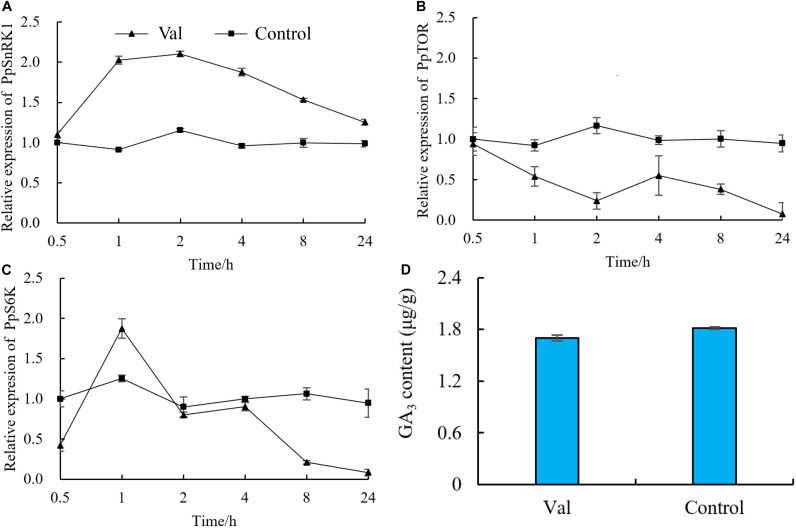
Effect of valine on relative expression of **(A)** *PpSnRK1*, **(B)** *PpTOR*, **(C)** *PpS6K* and **(D)** GA_3_ content in peach seedlings. Bars indicate means ± SE (*n* = 3). Different letters on the bars indicate significant difference between the treatments.

The expression of *PpSnRK1, PpTOR* and *PpS6K* was measured at different times following valine treatment. The expression of *PpSnRK1* increased significantly in valine-treated plants relative to control plants and increased its highest level at 2 h (Figure 7A). The expression of *PpTOR* decreased significantly in valine-treated plants relative to control plants and reached its lowest level at 24 h (Figure 7B). As a downstream gene of *TOR, S6K* is directly regulated by *TOR*. At 0.5 h after valine treatment, *PpS6K* expression decreased significantly in treated plants relative to control plants. It increased briefly at 1 and 2 h and then began to decrease again, reaching a value that was 91.2% lower than that of control at 24 h (Figure 7C). GA3 content in valine treatment was also lower than control at 48h (Figure 7D).

### Effect of Valine on the Enzyme Activity of AHAS and the Contents of BCAAs

**FIGURE 8:**
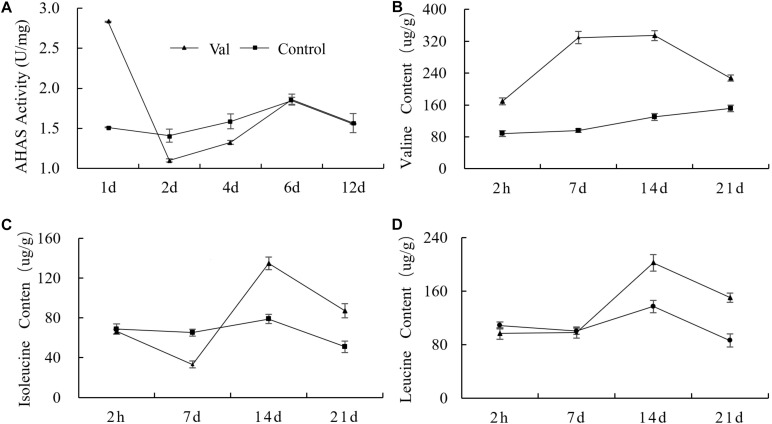
Effect of valine treatment on **(A)** AHAS enzyme activity and **(B–D)** the contents of three BCAAs. Each data point represents the mean (with SD bar) of three replicates.

Valine, leucine and isoleucine are called branched chain amino acids (BCAAs) because of their special branched structure. AHAS is the key enzyme in the first step of BCAA biosynthesis (Duggleby et al., 2008). After valine treatment, the activity of AHAS increased significantly at day 1 and was 87.1% higher than that of the control. At day 2 and 4, it began to decrease and was significantly lower than the control by 21.8 and 16.5%, respectively. After that, the activity of AHAS increased again and returned to the control level at day 6 and 12 (Figure 8A).

At 2 h after valine treatment, the content of valine in treated plants began to increase rapidly and reached a maximum at 7 days. It decreased after 14 days but remained significantly higher than that of the control plants throughout the entire measurement cycle (Figure 8B). Leucine content did not change significantly at 2 h or 7 days, but it was significantly higher in valine-treated plants at 14 and 21 days (Figure 8D). Isoleucine behaved differently than the other two amino acids. Its content was significantly lower in valine-treated plants at 7 days and increased gradually thereafter (Figure 8C). The contents of all three BCAAs were significantly higher in treated plants at 21 days.

### Isoleucine Can Alleviate Valine Inhibition and Restore Plant Growth

**FIGURE 9:**
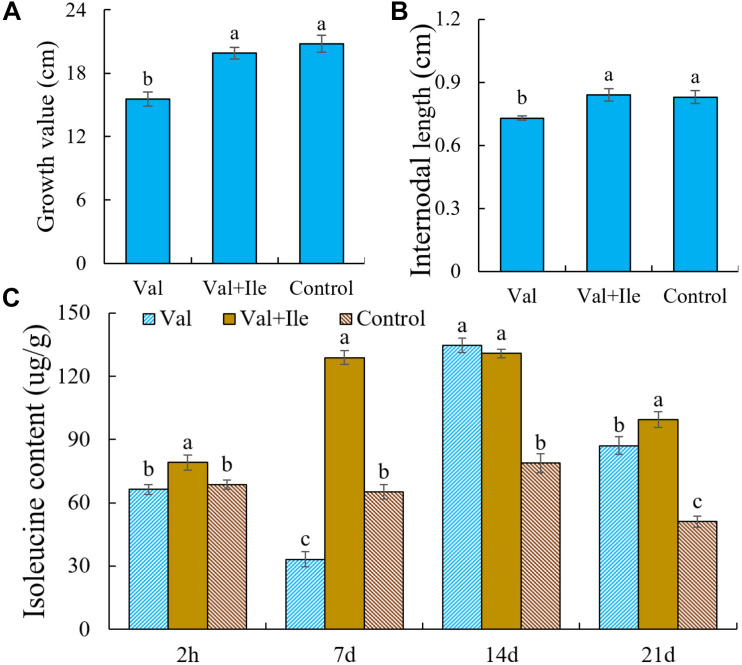
**(A)** Growth value, **(B)** internodal length, and **(C)** isoleucine content of peach seedlings sprayed with valine (Val), valine and isoleucine (Val + Ile) and water (Control). Bars indicate means ± SE (*n* = 3). Different letters on the bars indicate significant difference between the treatments.

Valine treatment is associated with increases in valine contents and short-term increases in leucine content, which may lead to feedback inhibition of AHAS activity and reduced isoleucine biosynthesis. We hypothesize that this disturbed balance of BCAA metabolism accelerates amino acid catabolism and energy consumption, resulting in reduced energy resources for new shoot growth. To further verify the above inference, isoleucine was added after valine treatment. There was no significant difference in plant height and internode length between this group and the control group. The content of isoleucine also increased significantly (Figure 9).

## Discussion

Bonner et al. (1992) showed that all amino acids except L-glutamine inhibit plant growth by inhibiting the synthesis of other amino acids. Forsum et al. (2008) found that plant growth was significantly inhibited under serine, valine, isoleucine, D-alanine, D-serine, D-arginine and D-valine treatments. Moreover, Jin et al. (2019) found that *bcat4* and *bcat5* genes knocked out rice mutants which were deficient in branched chain amino acid transaminase (BCAT) inhibited BCAAs synthesis and caused obvious growth inhibition. Furthermore, the growth of *E. coli* strain K-12 was inhibited by the addition of valine to growth media, and the addition of isoleucine relieved this growth inhibition (Leavitt and Umbarger, 1962).

Forsum et al. (2008) found that—in addition to glutamine and asparagine—other amino acids inhibited plant growth at a single high concentration or even inhibited plant growth by inhibiting the synthesis of other amino acids. Cui et al. (2014) showed that properly increasing the sprayed concentration of phenylalanine and tyrosine reduced the chlorophyll content and leaf area of honeysuckle leaves, thus inhibiting growth. In this study, phenylalanine, valine, and proline significantly inhibited the growth of peach seedlings, but leucine, isoleucine, serine and D-alanine did not confer any growth inhibition. In fact, leucine and isoleucine significantly promoted the growth of peach seedlings. Different results among studies may reflect different spraying times and plant varieties, which also have different requirements and capacities for the absorption and metabolism of amino acids (Wang, 2006). The root system is the main organ through which plants absorb and utilize underground nutrients. The root structure can change with changes in the internal environment (Li et al., 2010; Gao et al., 2016; Zhao et al., 2017), thereby affecting its ability to absorb and utilize nutrients (Yan et al., 2020). In this study, valine treatment either improved the total length, surface area, or had no significant effect on other indicators.

BCAAs are essential for human health as well as animal growth and development (Polis and Samson, 2020). For example, in the fish *Paralichthys olivaceus*, isoleucine is necessary for normal functioning of gill and intestinal tissue and enhances immune function (Wang et al., 2018). In various organisms, a lack of single BCAAs can cause reduced appetite (Nakahara et al., 2012; Deng et al., 2014) and an excess of BCAAs increases energy consumption (Choo et al., 1991; White et al., 2016). Plants and microorganisms are the main sources of BCAAs; animals cannot synthesize BCAAs, so BCAAs dietary consumption is necessary (Shaner et al., 1984). Previous studies have shown that AHAS is subject to BCAAs-mediated feedback inhibition (Pratelli and Pilot, 2014). As the target of herbicides, AHAS has been the focus of multiple research. Reduced AHAS activity leads to reduced synthesis of BCAAs, thus disturbing plant metabolism and potentially leading to reduced plant growth or survival.

Acetohydroxyacid synthase activity is controlled by substrate specificity and product feedback (Lee et al., 2011). Previous studies have shown that the response of AHAS to the three BCAAs is different in different microorganisms and plants (Harris, 1956; Holmberg and Petersen, 1988). In this study, the activity of AHAS initially increased (1 day) and then decreased (2–4 days) after spraying valine; it then returned to a normal level. This may be due to the lower increase in endogenous valine content at 1 day. In order to maintain the balance of amino acids, the plant increases the activity of AHAS and accelerates the synthesis of other amino acids. At 2 days, valine content increased continuously, and its catabolic rate was lower than its synthesis rate, which had a feedback inhibition effect on AHAS. Plants have the ability of self-regulation. With the extension of metabolic time, amino acids gradually return to equilibrium, and AHAS activity also returns to its original level. Spraying valine can increase the content of endogenous valine and leucine, feedback inhibit the activity of AHAS (Song et al., 2007; Zhao et al., 2015), and reduce the synthesis of isoleucine. At 7 days after valine treatment in this experiment, valine content increased, isoleucine content decreased significantly, and leucine content did not change, which may be due to plant variety differences (Cui et al., 2014).

Valine, as a signaling molecule, can be directly sensed by TOR protein. In this study, the expression of *PpSnRK1* increased, whereas *PpTOR* and its downstream target gene *PpS6K* decreased significantly in response to valine treatment. This may be because the concentration of valine was too high to inhibit the expression of *TOR*, or it may be because the energy in the plant was reduced, activating the *SnRK1* gene upstream of *TOR* and reducing its expression. The specific mechanisms require further study. In addition, previous studies have shown that the TOR signaling pathway is located upstream of the gibberellin–DELLA proteins signaling pathway. Inhibition of TOR activity can increase the expression level of DELLA proteins and affect the process of GA signal transduction (Zhang et al., 2018), and inhibition of the GA signal transduction pathway can effectively inhibit plant growth.

In our study, only valine caused significant peach seedling growth inhibition following treatment with individual BCAAs at the same concentration. Peach new shoot growth may be inhibited by: (1) Excess valine in plant tissue, which destroys the balance of amino acid metabolism, resulting in overall disturbed plant metabolism and inhibited growth (Song et al., 2011). (2) Low isoleucine content, which would subsequently affect the numerous metabolic processes that involve isoleucine, such as tissue repair and nitrogen metabolism, leading to an inhibitory effect on growth. (3) Excess valine in plant tissue, which leads to more vigorous amino acid metabolism and energy consumption, thereby depleting plant energy stores and inhibiting growth. The results described above are similar to those reported in animal-related studies (Coloso et al., 1999; Han et al., 2019). After valine treatment, the growth rate of peach tree new shoots did not decrease immediately, but began to exhibit growth inhibition at approximately 15 days after treatment. This might be because that valine and leucine replaced isoleucine in protein synthesis (Leavitt and Umbarger, 1962) during the early stages of valine treatment, which was sufficient to maintain the rapid growth of new shoots. Continued protein synthesis under isoleucine deficiency would ultimately cause a metabolic disorder and affect new shoot growth rate. However, because the plant is capable of self-regulation and repair, it remains viable. By the time normal amino acid metabolism gradually returned, the plant had shifted from vegetative growth to reproductive growth, so the new shoots no longer grew rapidly. By the time normal amino acid metabolism gradually returned, the plant had shifted from vegetative growth to reproductive growth, so the new shoots no longer grew rapidly. Following 60 days growth after valine treatment, which was the period of fruit expansion, the contents of N, P and soluble sugar were significantly higher than those in control. High N, P, and soluble sugar contents are beneficial for fruit development. Valine treatment caused increased single fruit weight and improved fruit quality appearance, whereas it had no significant effect on fruit shape index. Moreover, valine treatment significantly improved internal fruit qualities, such as soluble sugar, titratable acid, and Vc content. This may be because valine is used to regulate plant growth and as an additional supply of nitrogen at the same time (Hui et al., 2019). Nitrogen is closely related to the differentiation of fruit organs and the formation of tree structure. Nitrogen supplement can increase the photosynthetic rate, promote plant growth and improve fruit quality (Lone and Khan, 2007). Valine also inhibited shoot growth at the same time, which may be because valine regulated the vegetative and reproductive growth of plants, making more nutrients used for reproductive growth, thus limiting the vegetative growth of plants.

**FIGURE 10:**
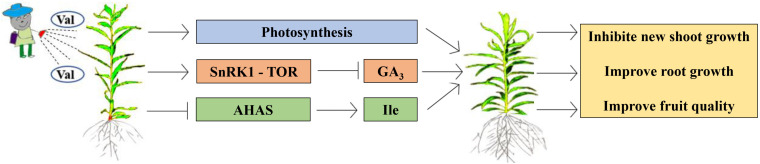
A working model for high concentration valine inhibits peach seedlings growth and improve fruit quality.

When the external environment or internal metabolism change, photosynthesis immediately responds (Tribouillois et al., 2015). As the main organ of photosynthesis, leaves play an important role in plant growth. They are very sensitive to internal and external changes. The above changes can be dealt with by changing leaf area, tilt angle, and length-width ratio (Jin et al., 2011). Changes in leaf morphology are related to structure and function. Generally, when the external morphology changes, the internal physiological activities also change (Guo et al., 2019). In this experiment, the peach tree new shoot growth and internode length following the spraying of valine of an appropriate concentration were significantly inhibited, which was not accompanied by negative effects on new shoot diameter, puncture strength, leaf morphology, or fruit quality. Further, based on the above research, we propose the circuit diagram (Figure 10).

## Conclusion

Different amino acids had different effects on the growth of peach seedlings. Phenylalanine, valine, and proline significantly inhibited peach seedling growth. Valine had the greatest inhibition effect, shortened internode length significantly, and had no adverse effects on shoot diameter or leaf and root growth. Compared with paclobutrazol, valine treatment improves peach tree fruit quality without reducing shoot diameter or puncture strength, and it does not affect leaf morphology. When the concentration of valine was 10 g⋅L^–1^, the endogenous valine content of peach seedlings was increased, AHAS activity was inhibited by feedback, isoleucine synthesis was decreased, the relative amounts of BCAAs were unbalanced, and growth was inhibited. In addition, a high concentration of valine increased the expression of *SnRK1*, and inhibited the expression of *TOR* and *S6K*, which made GA_3_ content reduced. In conclusion, valine is environmentally friendly to inhibit the growth of new shoots of peach trees by regulating the balance of *PpSnRK1* and *PpTOR* and the metabolism of branched chain amino acids and valine is conducive to the formation of peach fruit quality. Growing conditions could affect amino acid assimilation, whether valine inhibits the growth of peach shoots generally needs further study.

## Data Availability Statement

The original contributions presented in the study are included in the article/supplementary material, further inquiries can be directed to the corresponding author/s.

## Author Contributions

FP and YX conceived and designed the experiments. SL, QG, ZB, and YL performed the experiments. SL contributed reagents, materials, analysis tools, and wrote the manuscript. All authors contributed to the article and approved the submitted version.

## Conflict of Interest

The authors declare that the research was conducted in the absence of any commercial or financial relationships that could be construed as a potential conflict of interest.
